# Storage stability performance of composite modified asphalt with scrap non-tire automotive rubber, waste plastic pyrolytic oil and sulfur

**DOI:** 10.1371/journal.pone.0284473

**Published:** 2023-04-14

**Authors:** Ankush Kumar, Rajan Choudhary, Abhinay Kumar

**Affiliations:** 1 Department of Civil Engineering, Indian Institute of Technology Guwahati, Guwahati, Assam, India; 2 Department of Civil Engineering, Thapar Institute of Engineering and Technology, Patiala, Punjab, India; Beijing University of Technology, CHINA

## Abstract

Composite asphalt binder has emerged as a potential solution for improving asphalt functionality at a wide spectrum of temperatures. Storage stability of modified binder remains a main concern to ensure homogeneity during various stages including its storage, pumping, transportation, and construction. The aim of this study was to assess the storage stability of composite asphalt binders fabricated using non-tire waste ethylene-propylene-diene-monomer (EPDM) rubber and waste plastic pyrolytic oil (PPO). The influence of addition of a crosslinking additive (sulfur) was also investigated. Two different approaches were employed in the fabrication of composite rubberized binders: (1) sequential introduction of PPO and rubber granules, and (2) inclusion of rubber granules pre-swelled with PPO at 90°C to the conventional binder. Based on the modified binder fabrication approaches and the addition of sulfur, four categories of modified binders were prepared, namely sequential (SA), sequential with sulfur (SA-S), pre-swelled (PA), and pre-swelled with sulfur (PA-S). For variable modifier dosages (EPDM:16%, PPO: 2, 4, 6, and 8%, and sulfur: 0.3%), a total of 17 combinations of rubberized asphalt were subjected to two durations of thermal storage (48 and 96 hours) and then characterized for their storage stability performance through various separation indices (SIs) based on conventional, chemical, microstructural, and rheological analyses. The optimal storage stability performance was achieved at a PPO dosage of 6% under the four candidate approaches. It was also observed that the SIs based on chemical analysis and rubber extraction test had a good correlation with rheology-based SIs compared to the conventionally used softening point difference. A composite modified binder with PPO and EPDM rubber having adequate storage stability is a promising step in the use of sustainable composite-modified binders in asphalt pavement construction.

## Introduction

Asphalt binder derived from petroleum crude is a complex substance that is widely used in pavement engineering to bind stone aggregates in the base, binder, and wearing courses of flexible pavements. Owing to the drawback of being vulnerable to distresses induced by weather and temperature variations, poor aging resilience, and lower fatigue life, neat or unmodified binders may not satiate the demand of heavy loads of commuting traffic on the road [1]. Consequently, the synthesis and application of modified asphalt binders have been an important subject of interest in recent years. The utilization of granulated rubber crumbs obtained from discarded tire for modification of asphalt binder has been widely researched owing to benefits such as reduced temperature sensitivity, enhanced aging resistance and the pavement fatigue life. One of the main concerns associated with crumb rubber modified asphalt binder is its thermal storage stability or the compatibility of the base asphalt with the crumb rubber [2–4].

Rubber is used in many automotive components other than tires, including weather strips, hoses, vibration insulators, window and trunk seals, *etc*. The weight of these non-tire rubber parts accounts for 30% of the vehicle’s overall rubber weight, and ethylene-propylene-diene monomer (EPDM) rubber alone accounts for 50% of the non-tire automotive rubber [5]. A considerable quantity of scrap non-tire automotive rubber generates from discarded or scrapped automobiles, residues from the auto repair/service facilities along with different industries that manufacture these components. In recent studies, asphalt binders and mixes with EPDM rubber have shown good resistance to rutting at higher pavement service temperatures and a better moisture damage resistance [6–11].

On the other hand, various types of oil additives have also been found to improve asphalt binder performance, particularly at intermediate and low temperatures. Some commonly used oil additives include waste cooking oil, waste engine oil, soybean oil, and pyrolytic oils generated from wood chips, crop straw, sawdust, and waste tire [11–17]. Plastic waste generation has expanded in tandem with rising plastic use to the point where it is now a major worldwide problem in terms of its detrimental impact on the environment, human health, and the economy. Plastic wastes are non-biodegradable and can persist in the environment for a long time, and their increasing burden/buildup in landfills and water bodies is severely threatening aquatic and terrestrial animal life. Recycling and burning are two of the most commonly adopted plastic waste management options. Recycling plastics is difficult since it requires sorting the plastics in their types, which becomes a labor-intensive process, whereas burning plastics adds to hazardous and harmful pollutants in the environment [18]. In this direction, energy valorization processes such as pyrolysis are rapidly gaining popularity as an alternate means of plastic waste management. Plastic pyrolysis involves thermochemical conversion of longer-chain hydrocarbons into smaller ones in an oxygen-deficient environment at high temperatures, and the plastic pyrolytic oil (PPO) is generated as a co-product along with solid char and gases during the process [19]. This study addresses the application of PPO obtained from the pyrolysis of waste plastics in composite modification of asphalt binder with EPDM rubber.

In the composite modification of asphalt binder with oil and rubber, oil molecules may aid in enhancing the interaction between the rubber particles and the asphalt binder, which in turn may increase the rheological performance of the rubberized binders at a wider range of service temperatures. There are primarily two approaches to attempt the composite modification of the asphalt binder with rubber and oil: (1) sequential incorporation, wherein the two or more additives are introduced to the base binder in a sequence [9, 20–22], and (2) pre-treatment of the granulated rubber with the oil additive [11, 23–25] to graft oil molecules on rubber particles. The method used to prepare the composite rubberized binder can have a considerable impact on its rheological and thermal storage properties.

Phase separation or poor storage stability is a major concern with modified binders especially during its usage and thermal storage at elevated temperatures. The physical and chemical attributes of a modifier, such as its density, molecular weight, polarity, solubility, chemical structure, and reactivity, have a substantial influence on its homogenous distribution and compatibility with the base asphalt binder [26]. Similar to crumb rubber, granules of EPDM rubber can also have a propensity to induce phase separation by segregation from the asphalt phase during prolonged thermal storage [9–11]. Poor storage stability at elevated temperatures (mainly during storage, transportation and pumping of the binder through pipelines and during the production and construction of asphalt mixes) will likely to have undesirable repercussions on the performance of the modified binder and mixes [3]. Rubber granules may absorb light asphalt constituents after being added to the heated asphalt, which can cause the rubber to swell and increase in volume by up to three times [3]. The swelling of rubber, due to the absorption of light asphalt constituents, leads to changes in the microstructure of asphalt binder by altering its constituent proportions. It may also cause stiffening effect on the binder due to the inclusion of rubber particles with an increased volume [27].

Many solutions have been explored to alleviate the poor storage stability concerns of rubberized asphalt binders, including chemical and physical procedures. The chemical technique to improve the storage stability of modified binder entails the use of crosslinking additives such as sulfur, polyphosphoric acid, polyoctenamer, *etc*. Sulfur as a crosslinking agent is widely used with polymer- and rubber-modified asphalt binders as it has the ability to improve storage stability by forming covalent bonds among polymeric molecules and between hydrocarbons present in asphalt and the polymers, with the formation of sulfide and polysulfide chains between unsaturated carbon bonds (C = C and C≡C bonds) [3]. Physical procedures have been also suggested, including pre-treatment, surface activation, or composite modification using another modifier for making rubberized asphalt binder [2].

Kabir et al. [23] investigated the influence of five bio-oils (wood pellet, maize stover, waste vegetable oil, castor oil, and miscanthus) on interaction between asphalt and rubber by applying pre-treatment to graft molecules over the outer interface of the rubber granules. The wood-based bio-oil was reported to possess the highest association with rubber and the best rheological performance of all the examined bio-oils. The bio-oils also shown good absorption on the rubber surface and reduced the likelihood of the rubber to separate from binder. Ma et al. [24] added the waste cooking oil residue (WCOR) in rubber asphalt through simultaneous addition and pre-treatment approaches to investigate the role of the bio-oil on the compatibility and rheological performance of rubberized asphalt binder. The compatibility of the rubberized asphalt with pre-treated rubber with WCOR was found to be much better than that of the rubberized asphalt with untreated rubber granules and the rubberized asphalt made by simultaneous blending of rubber granules and WCOR with base asphalt. Lyu et al. [25] studied the effect of pre-treated rubber granules with waste cooking oil on the moisture resistance of bio-modified rubberized asphalt after thermal and ultraviolet aging. The pre-treated rubber with waste cooking oil proved to be a better modification approach for asphalt binder in terms of improved resistance to cohesive and adhesive damage. The enhancement was mainly credited to the function of oil modifier as a sacrificial component, inhibiting the interaction of free radicals with asphalt. Kumar et al. [11, 28, 29] found considerably improved microstructural, rheological, and compatibility performance through post thermal pre-treatment of non-tire automotive rubber in the presence of tire pyrolytic oil.

Although the effect of pre-treating rubber with some oil modifiers prior to addition with asphalt binder has been studied in a few studies, no specific study was found in the review on the pro-longed storage stability aspects of composite modified asphalt binders with EPDM rubber derived from non-tire automotive parts and plastic pyrolysis oil. Furthermore, no study is found to investigate the influence of sulfur as a crosslinking agent on the compatibility of PPO and non-tire automobile rubber composite modified asphalt binders.

**Fig 1:**
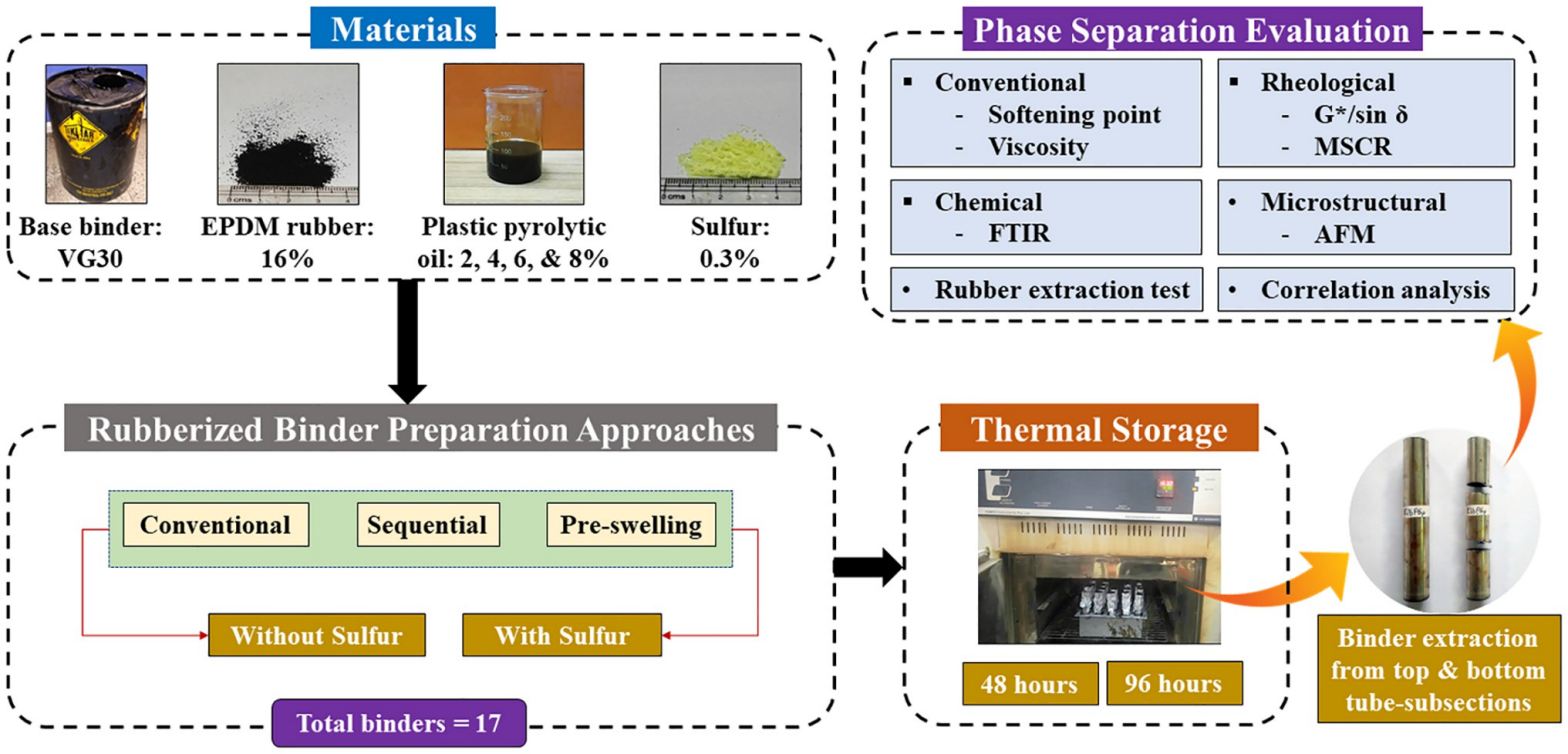
Outline of the research protocol adopted for this study.

Therefore, the main aim of this study is the storage stability characterization of the composite modified asphalt binders with non-tire automotive EPDM rubber and PPO obtained from the pyrolysis of waste plastics, prepared through sequential and pre-swelled/pre-treatment approaches with and without presence of a crosslinking additive (sulfur). The manuscript presents the results of physical, rheological, chemical, and microstructural investigations carried out to characterize the thermal storage stability performance of composite rubberized asphalt containing non-tire automotive EPDM rubber, PPO, and sulfur. Fig 1 depicts an outline of the research protocol adopted for this study. The composite asphalt was fabricated with a viscosity grade 30 (VG 30) binder as the base asphalt binder, EPDM non-tire automotive rubber (content: 16%), different dosages of PPO (contents: 2, 4, 6, and 8%), and sulfur as a crosslinking additive (content: 0.3%). The control and composite rubber modified binders were subjected to thermal storage at high temperatures for two durations of 48 and 96 h. Therefore, a total of 34 combinations of rubberized binders (17 binders × 2 thermal storage durations) were characterized for storage stability performance through physical, rheological, chemical, and microstructural investigations. The physical characterization was done using separation indices (SIs) based on softening point and viscosity, whereas the rheology-based SIs were based on Superpave rutting parameter (G*/sin *δ*) and multiple stress creep-recovery (MSCR) tests. Additionally, the Fourier transform infrared (FTIR) spectroscopy was employed to explore the variations in chemical functionality of the composite rubberized binders, and a SI based on the FTIR test was further developed to observe the storage stability performance. Atomic force microscopy (AFM) was used for microstructural investigation. A rubber extraction test was also carried out to further assess the efficacy of the different modified binder fabrication approaches (sequential and pre-swelling) having variable PPO dosages with and without sulfur.

## Materials and methodology

### Materials

**Fig 2:**
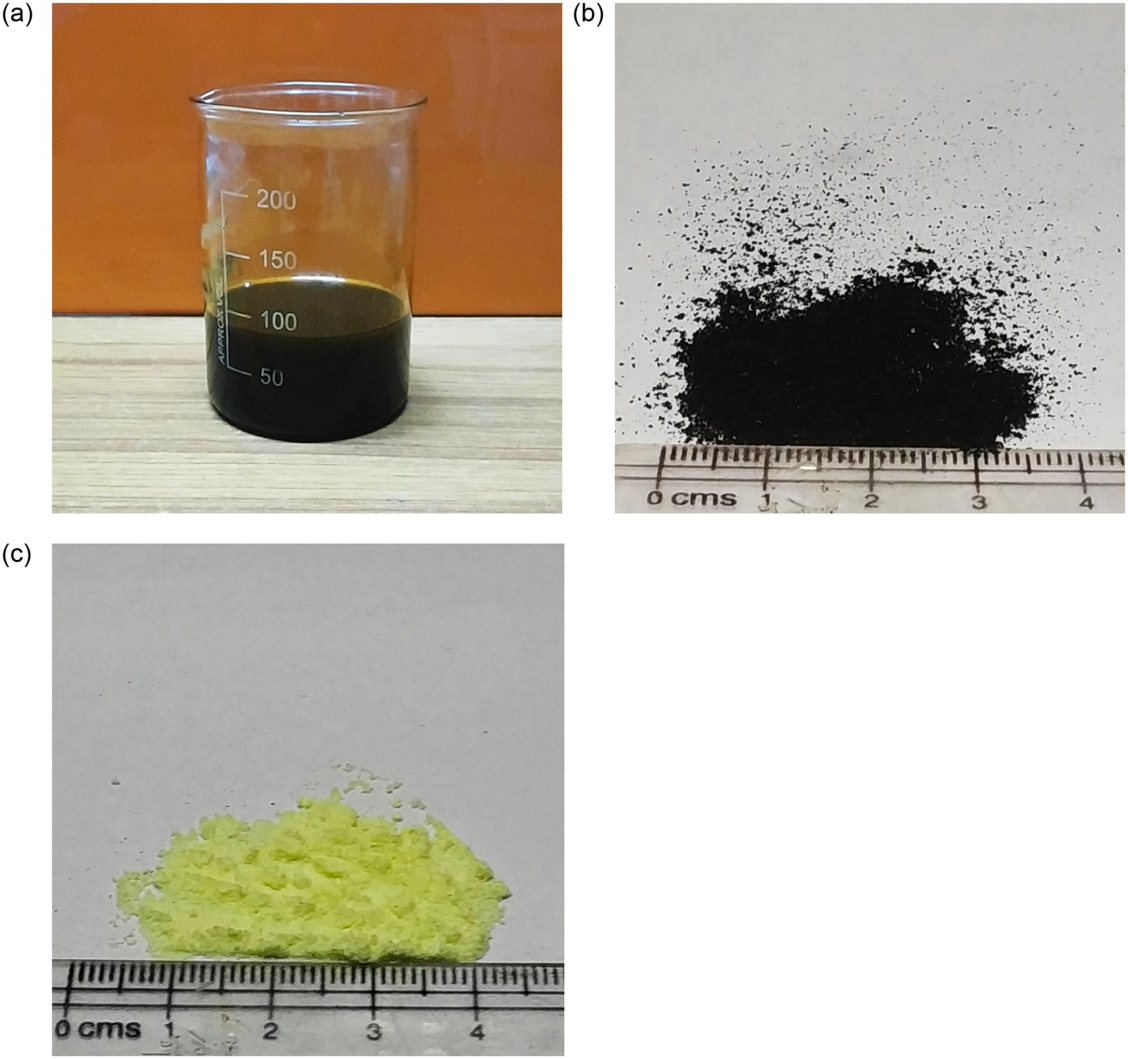
Pictorial representation of (a) non-tire automotive rubber particles, (b) PPO, and (c) sulfur.

The plastic pyrolytic oil (PPO) utilized was acquired from an industrial supplier after the pyrolysis of plastic wastes mainly composed of polypropylene. The pyrolysis procedure entailed loading the pyrolysis reactor with shredded plastic ranging in size from 15 to 25 mm in the presence of a proprietary catalyst. Nitrogen gas was injected into the reactor to eliminate all oxygen from the reactor in order to create an oxygen-deficient environment. The plastic fractured at around 250°C, and the vapors generated were condensed to obtain pyrolytic oil. The end results were primarily pyrolytic oil (70–80%), carbonaceous char (15–25%), and gases (10–15%). For an 8-ton batch, the entire procedure took around 15 hours. The PPO used in the study was unfractionated and yellowish-black in color, as shown in Fig 2a and had not undergone any further processing. The PPO had a viscosity of 6.4 mPa.s at 60°C and a density of 0.85 at ambient temperature (25°C). Non-tire automotive EPDM rubber granules used in the study were obtained from a commercial supplier/vendor of Delhi (India) after being mechanically shredded from the residuals of non-tire automotive rubber components collected from manufacturing establishments.

The size of the rubber granules affects the performance and storage stability of the rubber modified binder. Based on the literature [2, 30], rubber granules less than 300 μm (0.3 mm) in size (shown in Fig 2b) were employed in this study to prepare the rubber modified binders. A viscosity grade 30 (VG 30) binder was used as the base binder in this study for the production of rubber modified binders. The physical parameters of the base asphalt (without any modification) are shown in Table 1, and it complied with concerned Indian specification IS 73 [31]. Furthermore, sulfur powder (yellowish powder in physical appearance as shown in Fig 2c) was employed as a crosslinking additive in this study. Both sulfur and VG 30 were acquired from M/s TikiTar and Shell India Pvt. Ltd. (Gujarat, India).

**Table 1:** Physical characteristics of base binder.

Properties	Specification*	Result
Penetration at 25°C, 100 g, 5 s, 0.1 mm	*min* 45	52
Softening point, °C	*min* 47	54
Kinematic viscosity at 135°C, cSt	*min* 350	465
Absolute viscosity at 60°C, Poises	2400–3600	3407
Solubility in trichloroethylene, percent	*min* 99	>99
Flash point (Cleveland open cup), °C	*min* 220	280
***Tests on RTFO residue***		
Viscosity ratio at 60°C	*max* 4	2.3
Ductility at 25°C, cm	*min 40*	>100

*Requirements as per IS 73 [31].

### Preparation of rubber modified binders

For the preparation of control and composite modified binders through the sequential and pre-swelling approaches, rubber granules and sulfur (crosslinking additive) were used at a fixed dosage of 16% and 0.3%, respectively, while the PPO contents varied between 0 to 8% at an increment of 2% by weight of the binder.

#### Control rubber modified binder

**Fig 3:**
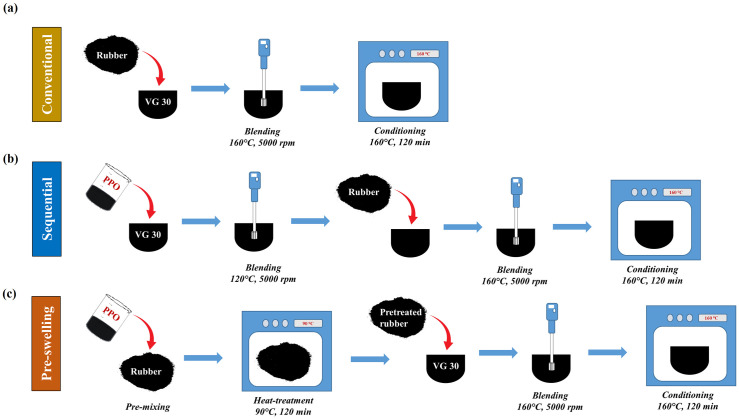
Schematic illustration for the formulation of composite asphalt binder.

Fig 3a depicts the formulation of the control rubber modified asphalt (designated as R). Firstly, the granulated rubber crumb was added to the base asphalt, which was preheated at 160°C, and then the blend was sheared and mixed at 160°C using a high shear mixer operating at 5000 rpm for a duration of 60 min. Thereafter, the prepared blend was subjected to thermal conditioning at 160°C for 2 h.

#### Sequential approach for composite rubber modified binders

Fig 3b outlines the procedure used for the formulation of composite rubber modified asphalt using the sequential approach (SA). The base asphalt was heated to 120°C, the required amount of PPO corresponding to different contents (2, 4, 6, and 8% by weight of binder) was added, and the combination was subsequently mixed for 20 min at 5000 rpm employing a high shear mixer. Subsequently, the desired amount of rubber (16% by binder weight) was introduced, and the prepared binder blend was mixed at 160°C for 60 min at 5000 rpm. Thereafter, the prepared composite rubberized binder was placed in an oven at 160°C for 2 h for conditioning. The composite rubber modified binders formulated with the SA method were designated as RP2, RP4, RP6, and RP8, respectively, for 2, 4, 6, and 8% dosages of PPO.

#### Pre-swelling approach for composite rubber modified binders

In the pre-swelling approach (PA) used to produce composite rubber modified binders (shown in Fig 3c), the PPO was first added to the rubber granules at the appropriate dosages to obtain pre-swelled rubber. The rubber pre-mixed with PPO was thereafter kept in an oven for 2 h of heat treatment at 90°C. Following this pre-swelling, the binder blending and conditioning operations followed were the same as for the control rubber modified binder (binder R). The composite rubber modified binders produced using the PA method were designated as RP2p, RP4p, RP6p, and RP8p for 2, 4, 6, and 8% dosages of PPO, respectively.

#### Composite rubber modified binders containing sulfur

The preparation of sulfur added composite rubber modified binders involved adding 0.3% dosage of sulfur to the produced binder formulation at 160°C, followed by 15 min of blending with a high shear mixer operating at 5000 rpm. The binders produced using the sequential approach with sulfur addition (SA-S) were classified as RP2-S, RP4-S, RP6-S, and RP8-S, whereas the binders prepared using the pre-swelling approach with sulfur addition (PA-S) protocol were assigned as RP2p-S, RP4-S, RP6-S, and RP8p-S. Table 2 details the nomenclature, formulation, and binder preparation methods employed in this study for all binders.

**Table 2:** Nomenclature and compositional information of studied asphalt formulations.

Binder	PPO, %	Rubber, %	Sulfur, %	Asphalt Formulation Method*
R	0	16	0	Conventional (Control)
RP2	2	16	0	SA
RP4	4	16	0	SA
RP6	6	16	0	SA
RP8	8	16	0	SA
RP2-S	2	16	0.3	SA-S
RP4-S	4	16	0.3	SA-S
RP6-S	6	16	0.3	SA-S
RP8-S	8	16	0.3	SA-S
RP2p	2	16	0	PA
RP4p	4	16	0	PA
RP6p	6	16	0	PA
RP8p	8	16	0	PA
RP2p-S	2	16	0.3	PA-S
RP4p-S	4	16	0.3	PA-S
RP6p-S	6	16	0.3	PA-S
RP8p-S	8	16	0.3	PA-S

*SA: Sequential addition of rubber and oil; SAS: Sequential addition of rubber and oil along with sulfur; PA: Pre-swelling with heat-treatment; PAS: Pre-swelling with heat-treatment along with sulfur.

### Thermal storage stability testing

The formulated binders of the study, including unmodified and composite rubber modified asphalt, were first simulated for the standard protocol for thermal storage stability in accordance with the ASTM D7173 [32] standard. A cylindrical, 25 mm diameter, 140 mm long metal tube having open top and closed base was used. The adequate amount of asphalt (50 g) was poured in the tube and the top end was closed with aluminum foil before conditioning in an oven at a high temperature of 163°C. All binders underwent two durations of thermal conditioning: 48 h (as per the ASTM D7173 [32] requirements) and 96 h (an enhanced duration considered for extended storage). Following this, both sets of tubes were refrigerated for 4 h at -10°C. The tubes were then cut into three equal-sized sections. Asphalt binder specimens were extracted from the top and bottom cut sections of the tube were then used for further phase separation or storage stability assessment.

### Physical and rheological analysis

Separation indices (SIs) based on different physical and rheological parameters were used to evaluate the thermal storage stability of the composite and control rubber modified binder samples derived from the top and bottom portions of the storage stability tubes. The physical tests included measuring the softening point using a ring and ball apparatus in accordance with IS 1205 [33] and the rotational viscosity of binders at 135°C with a Brookfield DV-II+ rotational viscometer following the procedure stipulated in ASTM D4402 [34]. Rheological parameters in the high-temperature domain were assessed with a 25 mm parallel plate geometry with a 2 mm gap between the plates on a dynamic shear rheometer (DSR). For high-temperature characterization of rubber modified binders with parallel plate geometry, a larger gap (2 mm compared with the traditionally employed 1 mm) is often advised to avoid the particulate effect of rubber granules on the results of rheological parameters [24, 35]. The high-temperature binder characteristics were evaluated at 64°C in terms of the Superpave rutting parameter (G*/sin *δ*) and the MSCR test. The G*/sin δ was computed using the complex modulus (G*) and phase angle (*δ*) measured with the application of 12% strain at 10 rad/s frequency. The MSCR test was carried out in accordance with ASTM D7405 [36] and the data from 10 cycles of creep and recovery by applying 0.1 kPa and 3.2 kPa stresses were ascertained. The strain outputs were computed with reference to the time for each creep-recovery cycle, which had a loading phase of 1 s and a 9 s recovery period. Eq. 1 was used to calculate the non-recoverable compliance (*J*_*nr*_), which is an indicator of the binder’s resilience to rutting.

$$J_{nr}=\frac{\varepsilon_{u}}{\sigma }$$ (1)

where, *ε*_*u*_ = unrecovered strain and *σ* = applied stress level (kPa) in each cycle.

### Chemical analysis

In this study, FTIR spectroscopy used attenuated total reflectance (ATR) approach to measure the chemical properties of binders by assessing absorbance peaks at different wavenumbers that correspond to specific functional groups. A total of 68 binders were analyzed for FTIR spectroscopy, representing the top and bottom segments of the 17 different rubber modified binders subjected to 48 and 96 h of thermal storage. The FTIR spectrum was analyzed between 400 to 2000 cm^-1^ wavenumbers. The asphalt sample was mixed with 10 percent weight-per-volume (w/v) tetrahydrofuran [(CH_2_)_4_O], and the spectrum was acquired after the solution was left to evaporate for 5 min.

### Microstructural analysis

The morphological appearance of rubber modified asphalt affects their functionality and aids in understanding the modalities of microstructural changes during thermal storage. The microstructure of the rubber modified binders exposed to 48 and 96 h of thermal storage was analyzed utilising AFM technique under tapping configuration. The binder samples for AFM analysis were prepared using the heat-cast method to provide a smooth and uniformly spread asphalt binder specimen for effective topographical analysis. A thin film of preheated rubber modified binder was placed onto a 12 × 12 × 2 mm glassware microscopy glass slide that was then placed for 5 min at 145°C in the oven to obtain a smooth sample layer on the glass slide. Thereafter, the glass slide with the thin film of binder was allowed to cool down and then placed in a sealed container before obtaining the AFM micrographs. The microstructural analysis of the specimens was carried out on a 20 × 20 μm scanning area at the ambient temperature (25°C).

### Rubber extraction test

**Fig 4:**
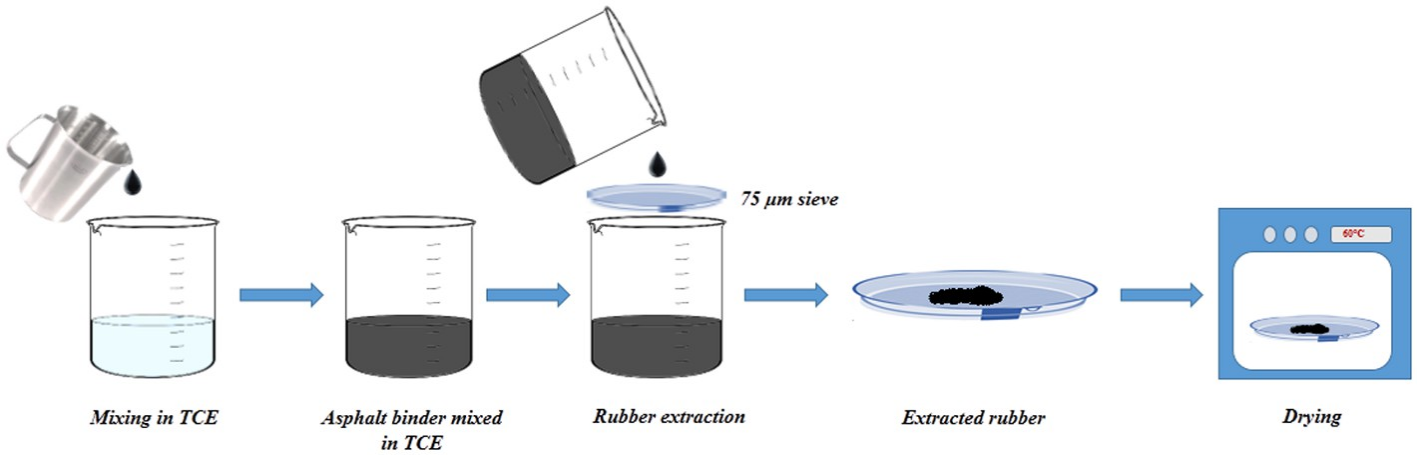
Schematics for rubber extraction test.

The deposition of non-dissolved crumb rubber granules to the bottom of the storage tank during thermal storage at high temperatures could cause phase separation in rubber modified asphalt binders. Extraction of non-dissolved rubber granules from rubber modified asphalt binders can be a viable approach for assessing phase separation occurrences. In this study, the non-dissolved rubber particles were extracted from the two tube subsections (top and bottom) after 48 and 96 hours of thermal storage. The rubber extraction procedure shown in Fig 4 is outlined as follows: A 10 ± 0.1 g of rubber modified binder extracted from sawed portions of the tube was dissolved in 100 mL trichloroethylene (TCE) in a container. As shown in the schematic of the rubber extraction test in Fig 4, the obtained solution of binder and TCE was filtered over a 75 μm mesh to remove the non-dissolved rubber granules in the binder. The residual rubber granules were afterward cleaned by washing with the fresh 50 mL TCE. The cleaned rubber granules were further placed for drying in an oven for 2 hours at 60°C to eliminate any presence of TCE. Finally, the dried rubber granules were collected and weighed to calculate the *SI*_*RET*_ based on the difference in mass of non-dissolved rubber particles from the top and bottom sections.

### Separation indices

The conventionally used separation index is in terms of softening point difference (SPD) of top and bottom binder samples extracted during the separation test (also quite often referred as cigar test) as per the ASTM D7173 Specifications. Many studies, however, have shown the inadequacy of the traditional separation index (SPD) in appropriately assessing the extent of phase separation in rubberized asphalts. Consequently, it has been suggested to assess the thermal storage stability based on separation indices measured from the rheological properties of binders, which are fundamental compared to empirically determine softening point [37, 38]. Therefore, three separation indices designated as *SI*_*Viscosity*_, *SI*_G*/sin δ_ and *SI*_*Jnr*_ were formulated with the proportion of viscosity at 135°C, G*/sin δ and MSCR *J*_*nr*_ at 3.2 kPa parameters measured at 64°C, respectively, on asphalt samples derived from the top and bottom tube sub-sections. Furthermore, two separation indices *SI*_*FTIR*_ and *SI*_*RET*_ were also determined based on FTIR and rubber extraction tests on the asphalt specimens removed from the top and bottom tube sub-portions. Therefore, this work utilized a total of six SIs (SPD, *SI*_*Viscosity*_, *SI*_G*/sin δ_, *SI*_*Jnr*_, *SI*_*FTIR*_, and *SI*_*RET*_) on all the 68 binder samples with the specifics of the expressions as presented in Table 3.

**Table 3:** Specifics of separation indices (SIs).

SIs Expression	Interpretation for improved storage stability
*SPD = Softening point difference of top and bottom sub-portions*	SPD = 0
$SI_{Viscosity}=\frac{Viscosity\;of\;top}{Viscosity\;of\;bottom}$	SI = 1
$SI_{G*/sin\;\delta }=\frac{G*/sin\;\delta \;of\;top}{G*/sin\;\delta \;of\;bottom}$	
$SI_{J_{nr}}=\frac{MSCR\;J_{nr}\;of\;bottom}{MSCR\;J_{nr}\;of\;top}$	
*SI*_*FTIR*_ *= Absorbance differential for binder from top and bottom sub-portions at 722 cm*^*-1*^ *wavenumber*	SI = 0
*SI*_*RET*_ = *Mass difference of extracted rubber from top and bottom tube sub-portions*	

## Results and discussion

### Physical tests

**Fig 5:**
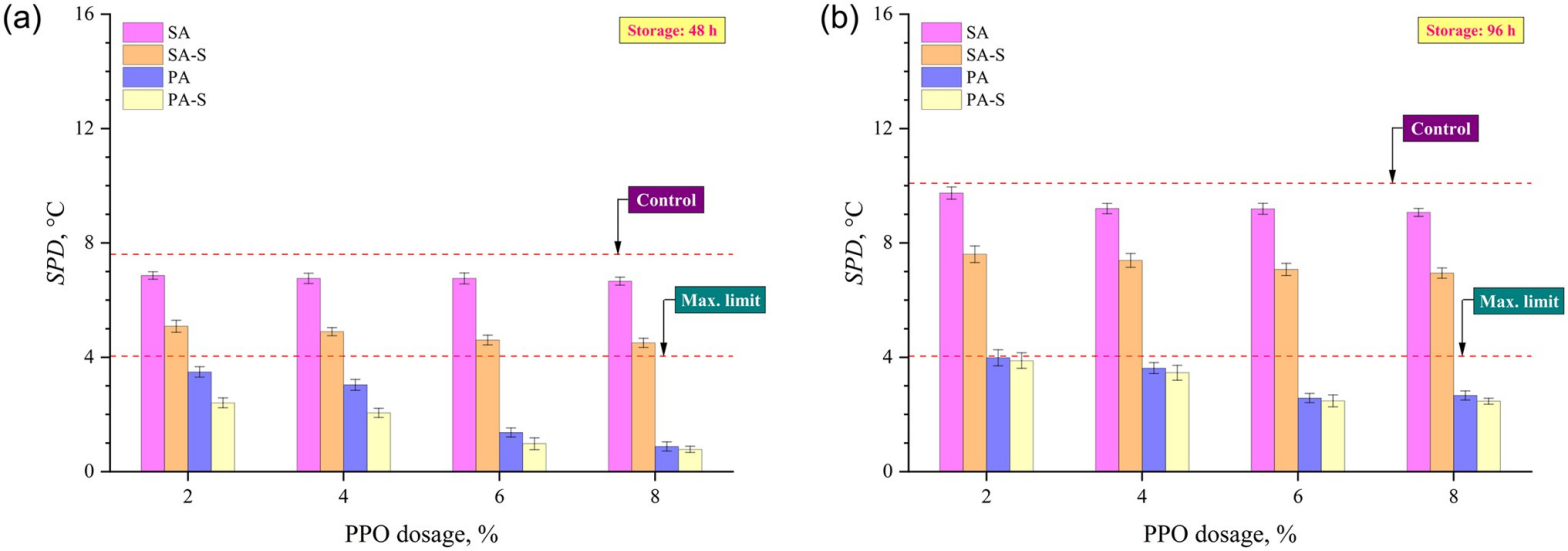
Softening point difference results after (a) 48 h and (b) 96 h of thermal storage.

Two separation indices based on conventional tests, SPD and rotational viscosity, were used to assess the extent of separation tendency of rubber modified composite and control binders. As presented in Table 3, SI based on viscosity is the ratio of binder viscosity from the top and bottom sections, and a *SI*_*Viscosity*_ value near 1 is preferred for better storage stability of the binder. On the other hand, SPD is the difference between the softening points of binders in the top and bottom sections, and IS 17079 [39] stipulates a maximum threshold of SPD of 4°C for a modified binder to display satisfactory storage stability, but the most ideal SPD value is 0. Fig 5a and 5b present the SPD results after 48 and 96 hours of thermal storage, respectively. It can be observed that binders showed a substantial increase in SPD as thermal storage duration increased from 48 to 96 hours. Binders produced with the subsequent addition of rubber and PPO in the SA method had relatively higher SPD values at both storage durations and also unable to achieve the stipulated SPD criterion of maximum 4°C at all PPO dosages. Although the SPD values reduced with the inclusion of sulfur, the SPD of asphalts prepared with the SA-S approach failed to satisfy the maximum permitted limit. For storage duration of 48 hours, the SPD reduced significantly for the binders with the pre-swelled and heat-treated composite of rubber and PPO (in PA method), and the application of sulfur (asphalts with PA-S method) generated the lowest SPD results at all PPO dosages. However, after the thermal storage for 96 hours, a slight increase in SPD was observed for the PA-S approach as compared to the PA approach.

**Fig 6:**
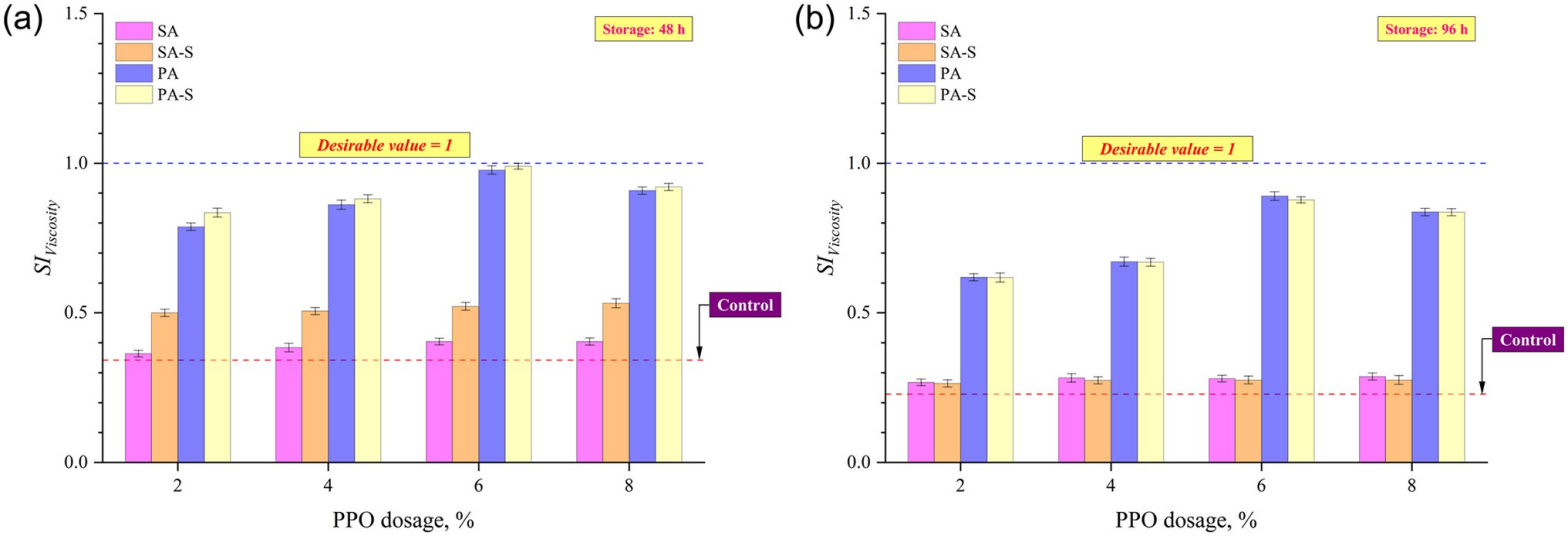
SI based on rotational viscosity after (a) 48 h and (b) 96 h of thermal storage.

The *SI*_*Viscosity*_ values after 48 and 96 h of thermal storage are shown in Fig 6a and 6b, respectively. At both storage durations, an incremental increase in *SI*_*Viscosity*_ values was found with increases in PPO dosages up to 6%, and thereafter slight reduction was witnessed in *SI*_*Viscosity*_. As shown in Fig 6a, after 48 hours of storage, the PA-S method produced the highest *SI*_*Viscosity*_ values at all PPO dosages, followed by PA, SA-S, and SA approaches. Furthermore, as storage time was increased to 96 hours, *SI*_*Viscosity*_ values for all binders declined in comparison to the values after 48 hours of storage (Fig 6b). The observed trends at each PPO dosage, however, were similar to those after 48 hours of storage. After 48 h of storage, all four approaches had the highest *SI*_*Viscosity*_ at 6% PPO dosage, with an increase of 18, 53, 186, and 190%, respectively, for SA, SA-S, PA, and PA-S binder preparation approaches as compared to the control binder (conventional rubber modified asphalt having 16% rubber and 0% PPO). In comparison to the binder R, the subsequent increase in *SI*_*Viscosity*_ at 6% PPO dosage after 96 h was 21, 19, 285, and 279 percent for SA, SA-S, PA, and PA-S methods, respectively. Considering the increase in storage duration, the *SI*_*Viscosity*_ values after 96 hours for 6% PPO content were 31, 47, 9, and 11% lower for SA, SA-S, PA, and PA-S approaches, respectively, when compared with subsequent *SI*_*Viscosity*_ values of binders after 48 hours of storage. When comparing conventional separation indices based on SPD and *SI*_*Viscosity*_, sulfur as a crosslinking agent had a significant effect on SPD even after 96 hours of storage, whereas its impact on *SI*_*Viscosity*_ was noticeable for binders subjected to the storage of 48 h and had minimal effect on *SI*_*Viscosity*_ of binders stored for 96 hours.

### Rheological tests

**Fig 7:**
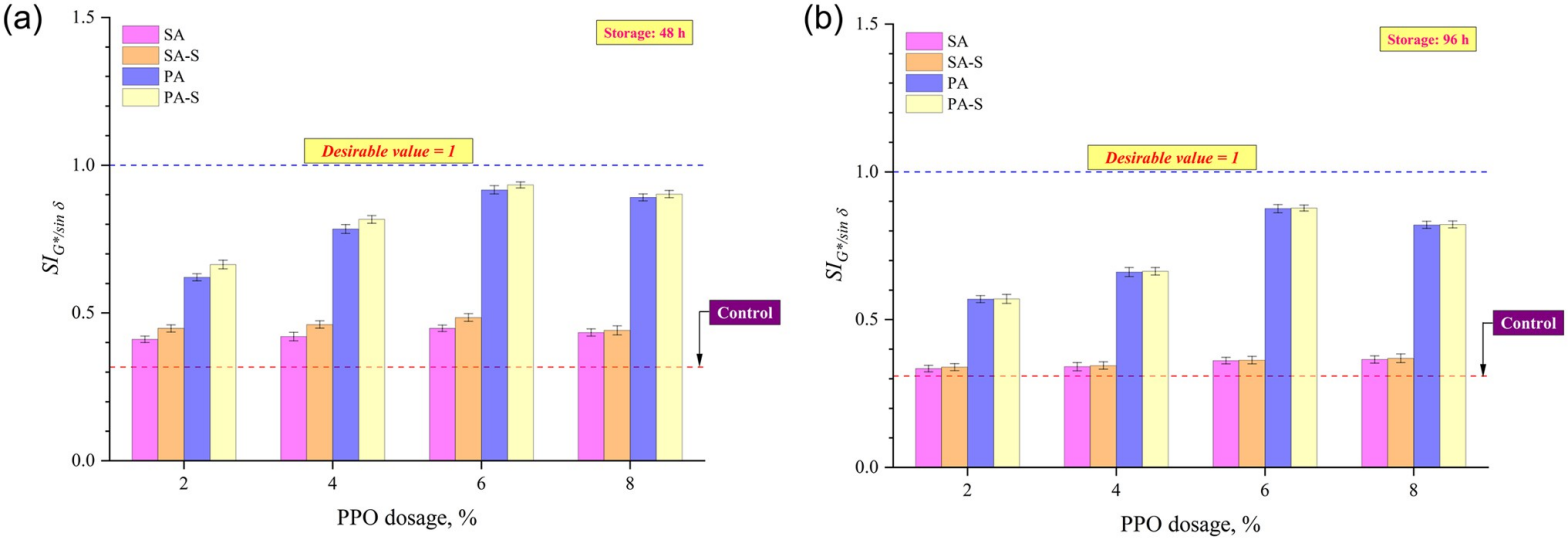
SI based on G*/sin *δ* after (a) 48 h and (b) 96 h of thermal storage.

**Fig 8:**
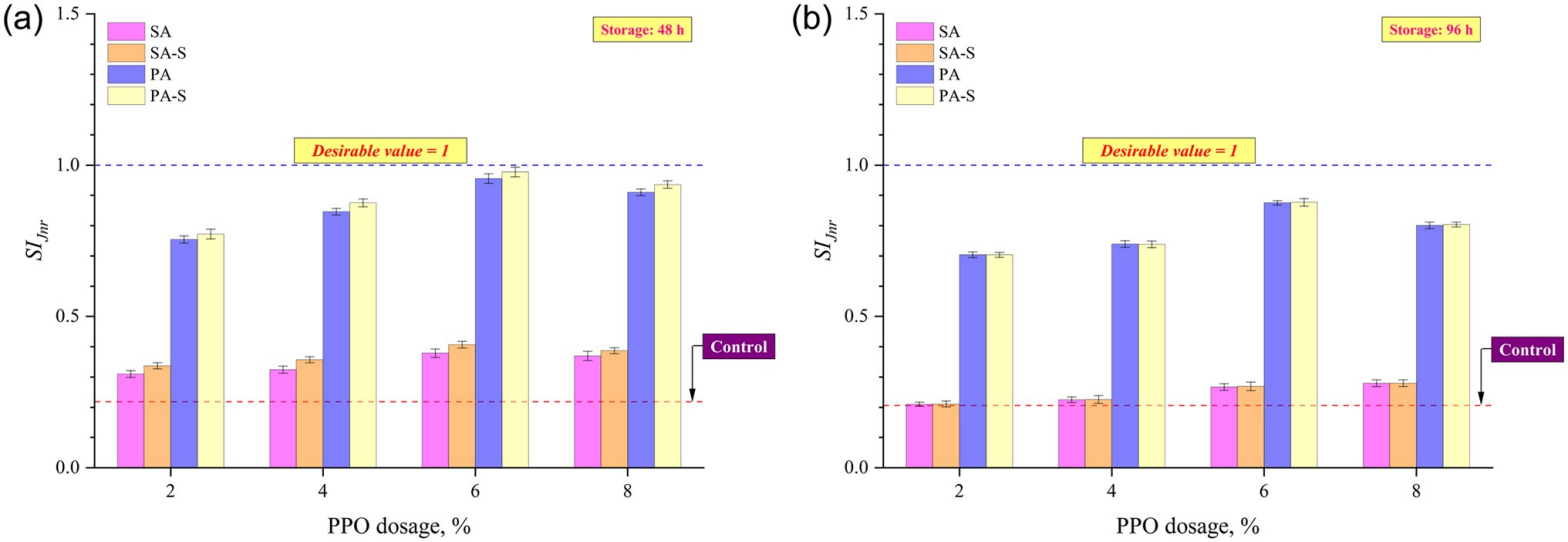
SI based on MSCR *J*_*nr*_ after (a) 48 h and (b) 96 h of thermal storage.

Rheological separation indices based on G*/sin δ and MSCR *J*_*nr*_ at a stress level of 3.2 kPa were considered in this study. The SI derived using G*/sin δ was the proportion of values for asphalt from two tube sub-segments, but the SI computed using MSCR *J*_*nr*_ was the proportion of bottom section to top. A SI around 1 is preferable since it implies that the binders from the top and bottom parts of the storage tube have similar characteristics. Figs 7 and 8 show that the binder R (rubber = 16%; PPO = 0%; sulfur = 0%) had the lowest SIs after 48 and 96 hours of storage, implying that the introduction of rubber particles alone to the base asphalt binder possess storage stability issues. This indicates that rubber granules at an elevated temperature (163°C) plunge to the bottom of the storage stability test tube during long-term storage. The SIs of all four asphalt formulation methods augmented with an increase in PPO content up to 6%, followed by a marginal decrease at 8% PPO content. Considering the binders after 48 hours of thermal storage, the SIs ranked as SA<SA-S<PA<PA-S among the four asphalt formulation methods. Figs 7a and 8a illustrate the substantial improvement in SIs after the application of crosslinking agent (sulfur) to the asphalt matrix, as SA-S and PA-S approaches had higher SIs than SA and PA approaches, respectively. The effect of sulfur on SIs disappeared to a large extent after prolonged storage for 96 hours since SA and SA-S had very close SIs, as did PA and PA-S (Figs 7b and 8b). However, pre-swelling approaches (PA and PA-S) still had higher SIs than sequential approaches (SA and SA-S), implying the effectiveness of the pre-swelling approach in enhancing the thermal storage stability even after pro-longed period of storage.

The notable enhancement in storage stability for asphalts produced by the PA method can be ascribed to the pre-swelling of granulated rubber crumb owing to the mixing of PPO with rubber along with the application of heat treatment to the rubber and oil composites. It can be asserted that the pre-mixing of PPO and rubber coupled with heat treatment aids in the acceleration of the swelling operation by integrating light-weight components of PPO into the rubber granules. With an appreciable rubber swelling it creates the premise for the subsequent disintegration of granulated rubber crumbs in the binder matrix. This allows rubber particles to disperse more homogeneously in the asphalt phase resulting in an improvement in the stability of asphalt during thermal storage. The improved storage stability following the incorporation of sulfur in both sequential and pre-swelling strategies is ascribed to the crosslinking effect, wherein the sulfur particle binds polymer chains together as well as binds polymers together with binders. The ineffectiveness of sulfur on SIs after prolonged storage (96 hours) can be attributed to the disappearance of crosslinking networks due to the destructive effect of aging imparted by storage at an elevated temperature (163°C) for longer duration [40–42].

### Chemical test

**Fig 9:**
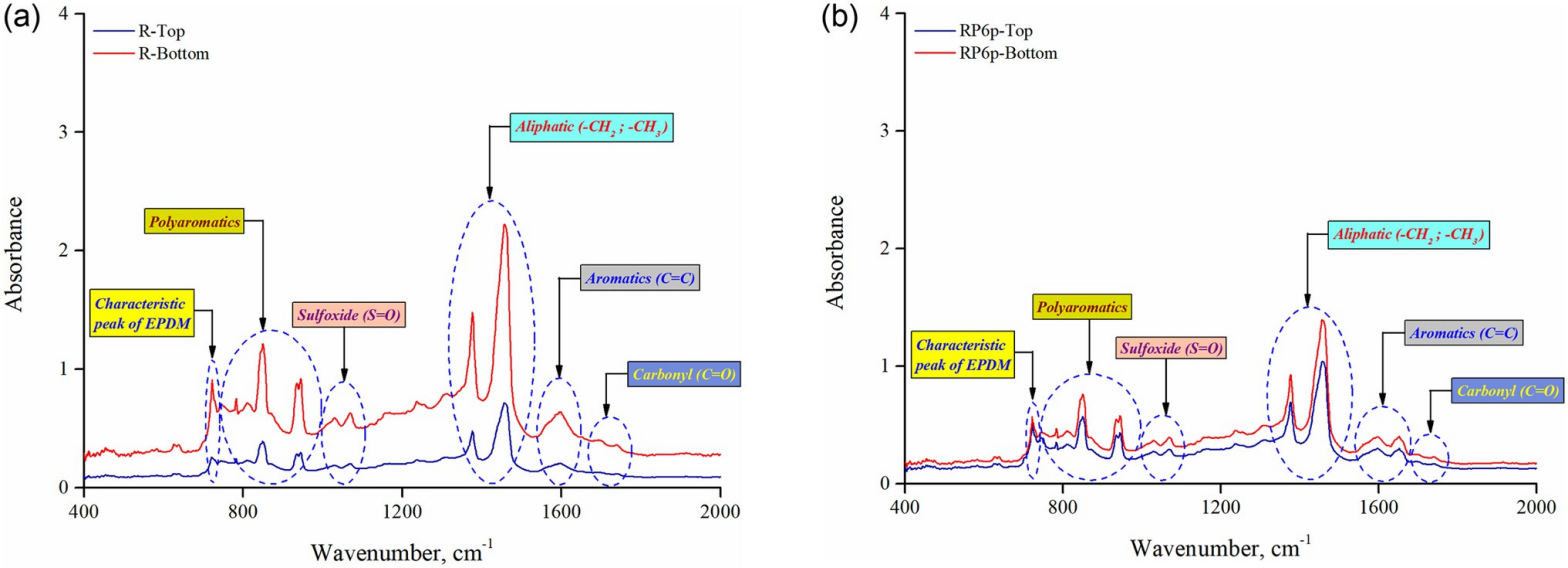
FTIR spectra of top and bottom parts of (a) binder R and (b) binder RP6p.

Fig 9a and 9b illustrate the FTIR spectra of asphalt specimens extracted from the tube sub-sections of binders R and RP6p, respectively, for wavenumbers 400 to 2000 cm^-1^. The spectral peak around 1670 to 1725 cm^-1^ belongs to C = O carbonyl functional groups, while absorbance peaks between 1535 to 1625 cm^-1^ correspond to aromatics with C = C stretching [43–46]. The aliphatic group with saturated C-H bending vibration was ascribed to the two prominent spectral peaks between 1350 and 1510 cm^-1^. The occurrence of sulfoxide (S = O) entities was responsible for the identified absorption bands between 1010 and 1070 cm^-1^. The occurrence of absorbance peaks between 790 to 970 cm^-1^ pertains to the C = C bending of polyaromatics. The strong spectral peaks near 850 and 945 cm^-1^ belong to the trisubstituted and disubstituted alkenes, respectively [46]. In the spectrum, the peak at 722 cm^-1^ corresponds to the idiosyncratic absorption band of EPDM, ascribed to the existence of ethylene sequences in the EPDM backbone, and is mainly caused by rocking vibrations of the CH_2_ typically of (CH_2_)_n_― type where n ≥ 5 [47–49]. There is no distinctive difference in the spectrum of binders from the tube sub-sections in both R and RP6p binders (Fig 9a and 9b), except for an absorbance peak at 1651 cm^-1^ corresponding to aromatics.

The spectral curve of binder R (control binder) exhibits higher absorbance intensities for the bottom section than in the top part, as shown in Fig 9a. Meanwhile, the spectrum profiles for the bottom and top sections of the RP6p binder are fairly nearer in Fig 9b. This can be ascribed to the increased compatibility of the binder with a relatively uniform dispersion of pre-swelled rubber granules throughout the storage tube. Zhang et al. [50] derived a similar deduction after observing a relatively close spectrum curve for the two sub-sections of a bio-asphalt during storage at elevated temperature. Lu et al. [51] used distinctive spectral bands at 965 and 700 cm^-1^ wavenumbers to assess phase segregation in SBS modified asphalt binder (closely correlated to polymer concentration). It was noted that the variation in absorption intensity at these peaks could be used to predict phase separation. Similarly, this study analyzed the absorption intensity at 722 cm^-1^ (idiosyncratic band of EPDM) to estimate its thermal storage stability. As mentioned in Table 3, *SI*_*FTIR*_ is defined as the differential in absorption intensity at 722 cm^-1^ for binders from the storage tube sub-sections, with ’0’ being the desired value for an ideal storage stable binder.

**Fig 10:**
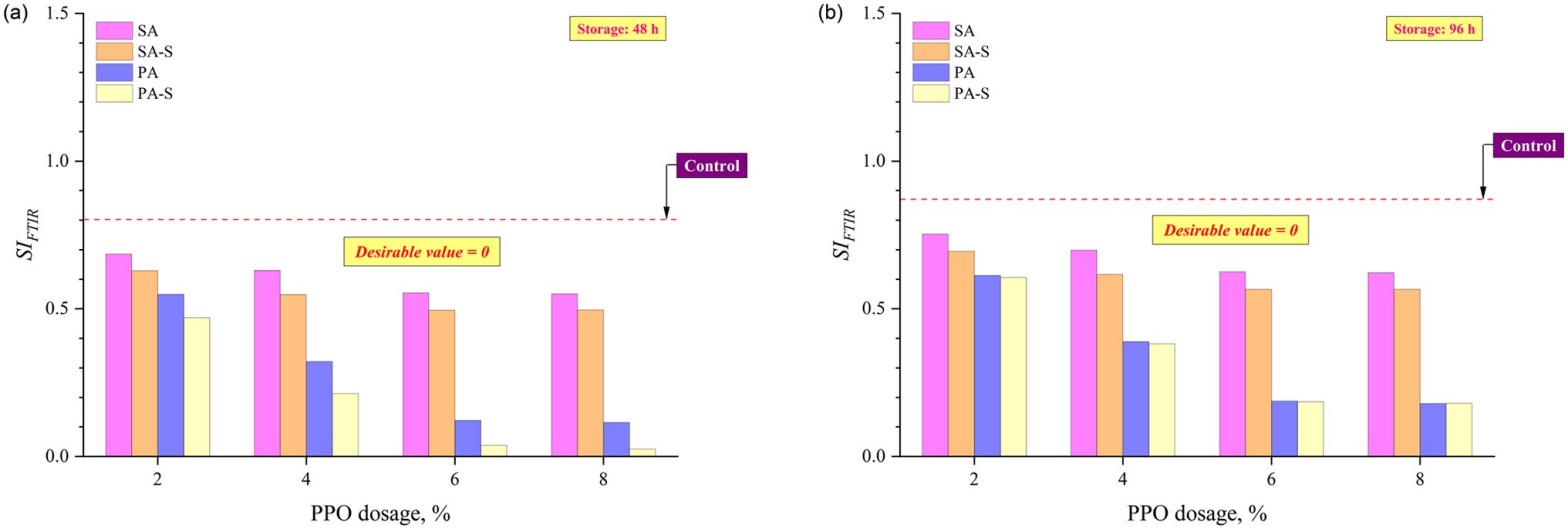
Chemical separation index (*SI*_*FTIR*_) after (a) 48 h and (b) 96 h of thermal storage.

**Fig 11:**
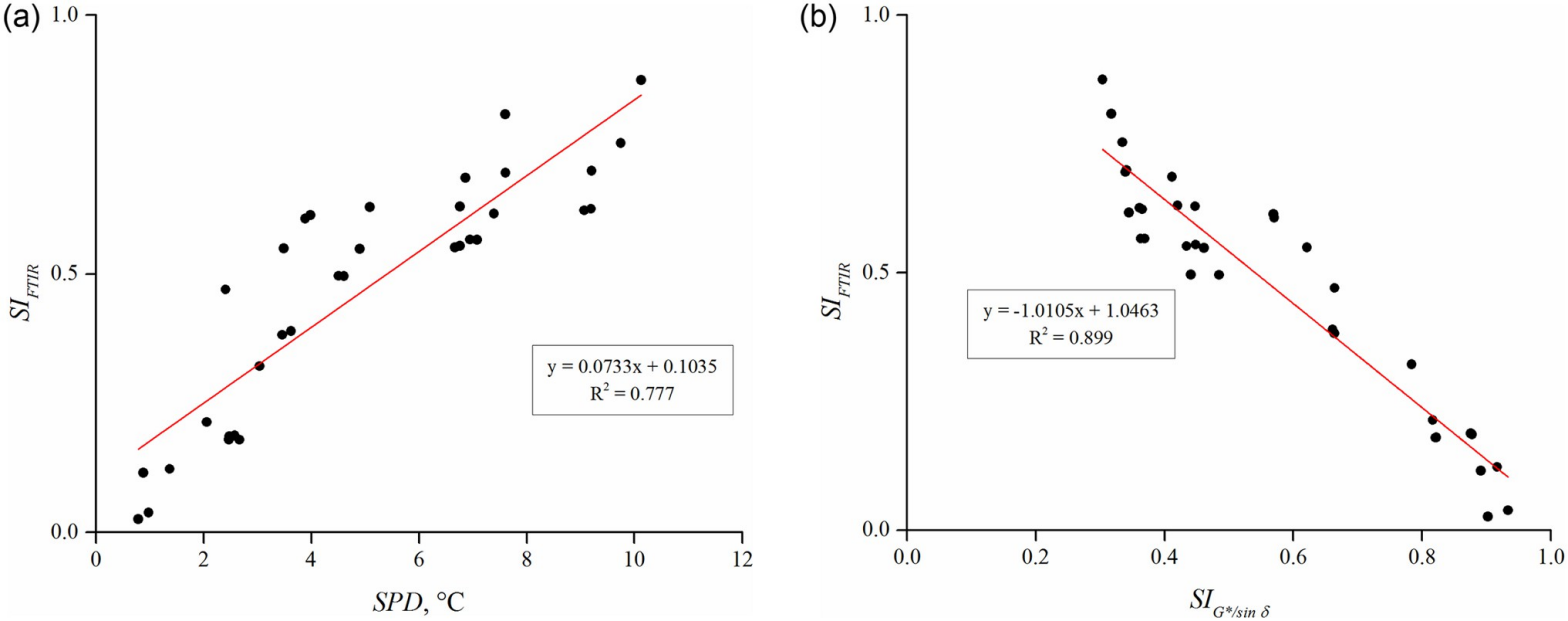
Chemical separation index (*SI*_*FTIR*_) as a function of (a) SPD and (b) *SI*_G*/sin δ_.

Fig 10a and 10b present the result for *SI*_*FTIR*_ for binders subjected to 48 and 96 hours of thermal storage. The *SI*_*FTIR*_ decreased with an increment in PPO dosage and also with a shift in binder preparation approaches such that PA-S had the lowest *SI*_*FTIR*_ values, followed by PA, SA-S, and SA. This trend was consistent even after 96 hours of storage, implying the efficacy of PPO and pre-swelling approaches in improving the storage stability of rubber modified binders. The lower value of *SI*_*FTIR*_ indicates a smaller difference in absorbance intensity of binders from the top and bottom sections at the characteristic peak of EPDM rubber corresponding to 722 cm^-1^, and a more homogeneous rubber dispersion in the binder matrix is responsible for this reduction. Fig 11 illustrates the correlation between separation indices based on conventional (*SPD*), rheological (*SI*_G*/sin δ_), and chemical (*SI*_*FTIR*_) evaluation of binders. The co-efficient of determination was 0.777 between SPD and *SI*_*FTIR*_ (shown in Fig 11a), while 0.899 between *SI*_G*/sin δ_ and *SI*_*FTIR*_ (shown in Fig 11b), indicating a better correlation of separation index based on chemical analysis with rheological evaluation than the conventional softening point difference approach. Moreover, the fair correlation coefficients indicate that the formulated chemical separation index is effective in assessing the extent of segregation between the rubber particles and the asphalt binder.

### Microstructural test

**Fig 12:**
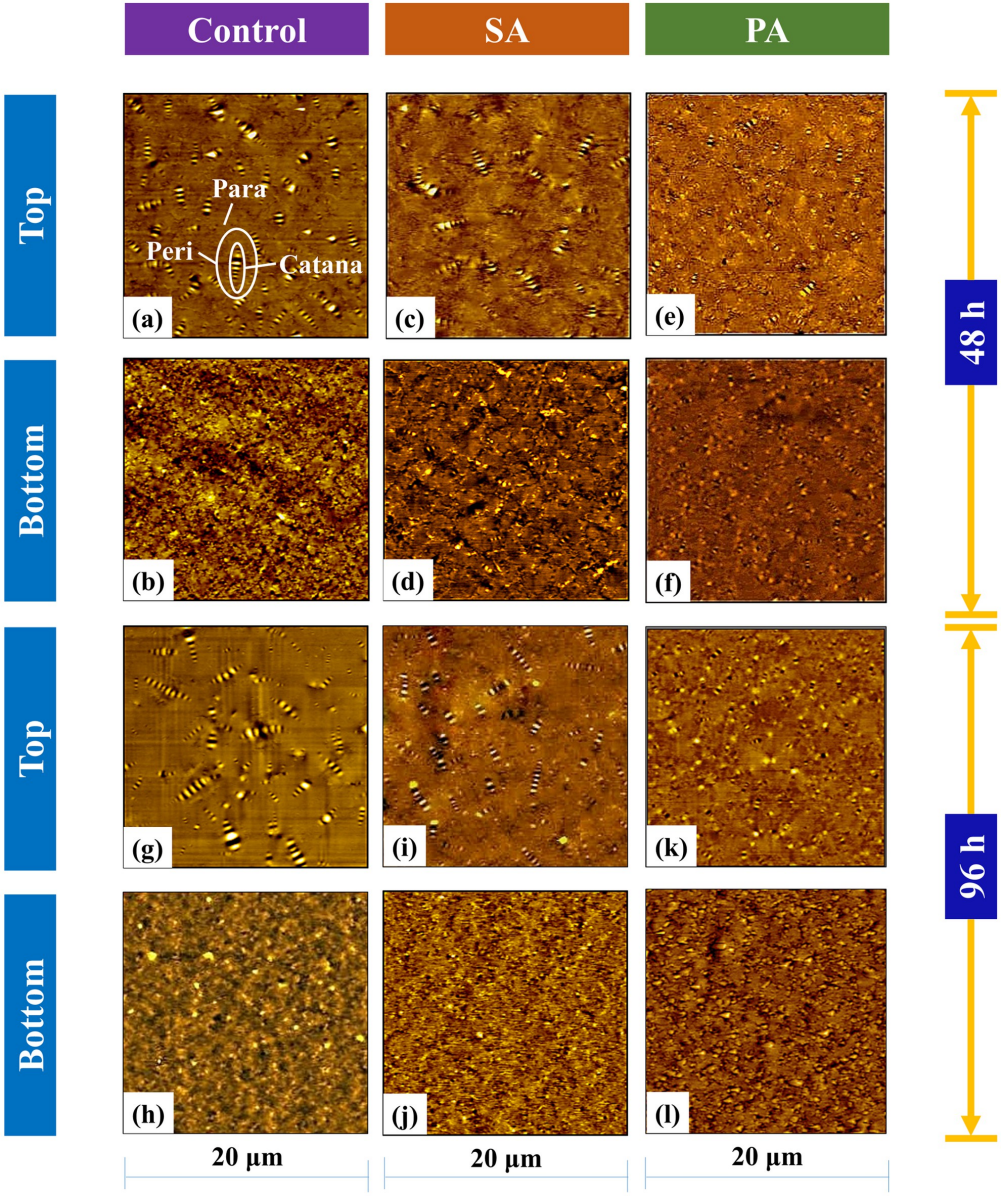
AFM micrographs after 48 h of thermal storage of binders: (a) R-top, (b) R-bottom, (c) RP6-top, (d) RP6-bottom, (e) RP6p-top, (f) RP6p-bottom; and after 98 h of thermal storage of binders: (g) R-top, (h) R-bottom, (i) RP6-top, (j) RP6-bottom, (k) RP6p-top, (l) RP6p-bottom.

The micromorphology of the asphalt obtained from the two sub-sections of the storage tube was analyzed using the AFM micrographs. As illustrated in Fig 12, topographic scans of samples revealed catana domains (also known as bee structures) enclosed by peri domains and distributed throughout the para domain. The rubber modified binders show smaller and indistinctive catana domains in the topography due to the presence of rubber particles [52]. Furthermore, Liu et al. [53] interpreted phase segregation occurring in polymer-modified binders considering dissimilarity in catana domain dimensions. For control binder R, the top section (Fig 12a) had a discrete microstructure with catana domains (bee structures) of varying sizes, but the bottom portion (Fig 12b) had smaller catana domains owing to the existence of more rubber particles in that portion. The microstructure of the asphalt from the two tube sub-sections for the SA method (binder RP6) was still distinctively dissimilar after the addition of PPO, as seen in Fig 12c and 12d, whereas Fig 12e and 12f displayed relatively similar microstructural topography of top and bottom sections with smaller catana domains for the composite binder prepared with PA approach (binder RP6p), suggesting an enhanced homogeneity of the pre-swelled composite rubberized binder. Fig 12e and 12f present indistinct boundaries of peri and para domains and a reduction of contrast between catana and peri domains in the topographic images. The greater dissimilarities between topographic images from the top and bottom parts of binders extracted after 96 hours of thermal storage can be attributed to the combined influence of a higher amount of rubber particles present in the bottom section and higher degree of aging of binders owing to the longer thermal storage duration. However, the PA approach still had relatively higher similarity in dimensions of catana domains for the top (Fig 12k) and bottom (Fig 12l) sections due to increased storage stability of composite rubberized binder owing to pre-swelling.

### Rubber extraction test and correlation analysis

**Fig 13:**
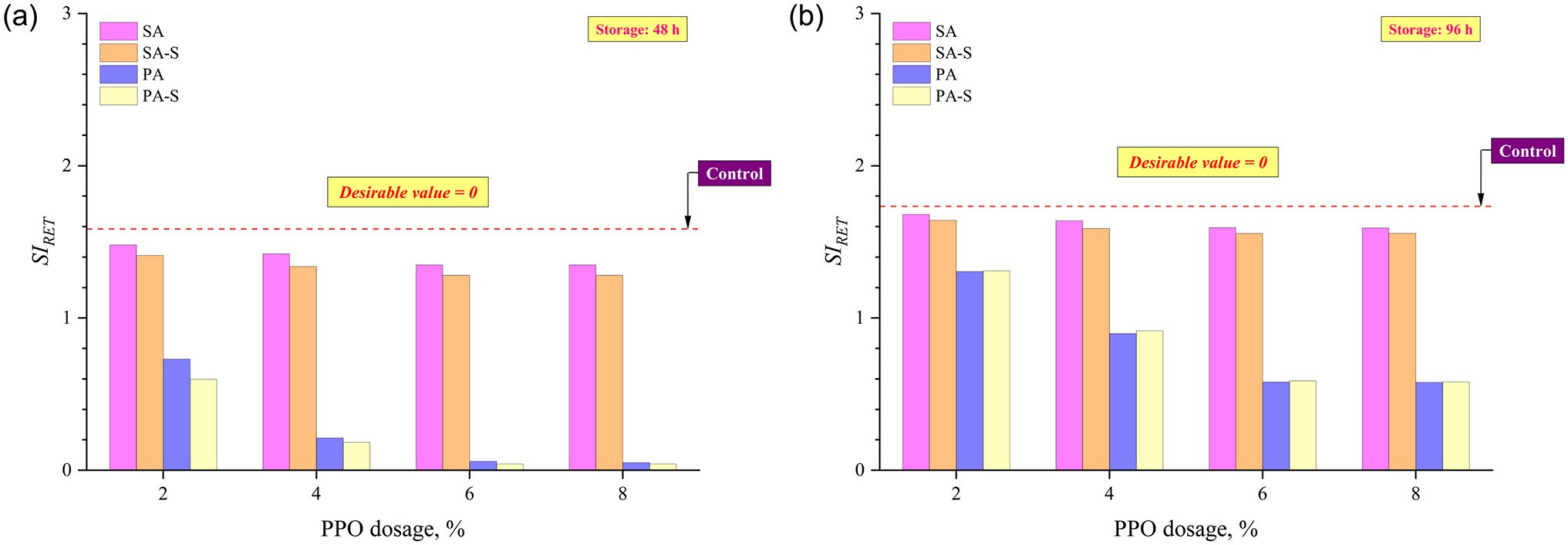
*SI*_*RET*_ results from rubber extraction test after (a) 48 h and (b) 96 h of thermal storage.

**Fig 14:**
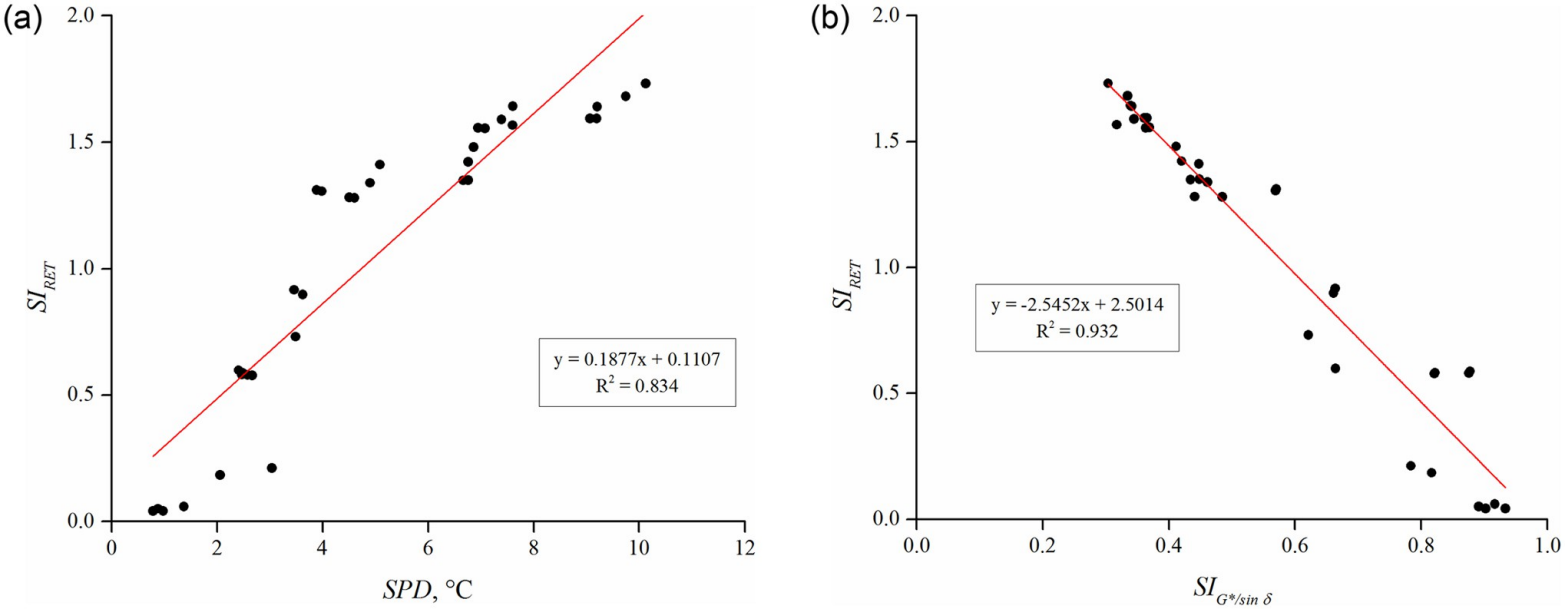
*SI*_*RET*_ as a function of (a) SPD and (b) *SI*_G*/sin δ_.

In order to analyze the effect of PPO content, binder preparation approaches (sequential and pre-swelling), and crosslinking agent (sulfur) on the phase separation or thermal stability of rubberized binders, the rubber extraction test (RET) was performed to compute the *SI*_*RET*_ as the mass difference of extracted undissolved rubber particles from the top and bottom rubberized binders after thermal storage. An ideally storage stable binder will exhibit *SI*_*RET*_ value of 0, representing an equal amount of extracted rubber from the top and bottom portions. Fig 13 shows that the *SI*_*RET*_ decreases with an increment in PPO dosages for both 48 and 96 hours of thermal storage. At each PPO dosage, in terms of binder preparation approaches, *SI*_*RET*_ can be ranked as SA > SA-S > PA > PA-S for 48 hours of thermal storage (Fig 13a); however, this trend changed as SA > SA-S > PA-S > PA when stored for 96 hours at temperature (Fig 13b). The higher *SI*_*RET*_ results for PA-S than the PA approach (shown in Fig 13b) indicate that the beneficial effect of sulfur on storage stability may reduce with an increase in the duration of thermal storage. Moreover, it can also be inferred that the positive effect of pre-swelling remains prevalent on storage stability even after 96 hours of thermal storage. Fig 14 depicts the relationship between separation indices based on SPD, *SI*_G*/sin δ_, and *SI*_*RET*_. The co-efficient of determination between SPD and *SI*_*RET*_ was 0.834 (shown in Fig 14a), whereas it improved to 0.932 between *SI*_G*/sin δ_ and *SI*_*RET*_ (shown in Fig 14b), implying that *SI*_*RET*_ has a better correlation with rheologically determined *SI*_G*/sin δ_ than SPD.

### Mechanism of phase separation

An asphalt binder is a complex substance made up of a large number of molecular species with significantly varying physical and chemical characteristics. The colloid theory of asphalt structure describes an asphalt binder as a system made up of high-molecular-weight components (asphaltenes) dispersed in a low-molecular-weight continuous phase (maltenes). Consequently, maltenes provide the solvating power necessary to keep asphaltenes peptized/deflocculated with adequate mobility [54]. Adding rubber modifiers to the heated asphalt causes the rubber particles to absorb maltenes and swell up, thus decreasing the amount of light fractions in the asphalt and thereby reducing its solvating capacity [37]. Introducing rubber with higher molecular weights or densities may therefore disturb the dynamic equilibrium in asphalt, and an overall reduction in the homogeneity of the asphalt matrix.

During thermal storage, the gravitational field-induced deposition of rubber granules occurs along with the impacts of thermodynamics, the asphalt system becomes unstable and results in a phase separation, which can be observed from the evaluation of the binders from top and bottom region of the storage tube during separation test. To minimize the phase segregation propensity of rubberized asphalt, the deposition pace of granules should be lowered, along with the efforts to limit deposition velocity by lowering the density differential between rubber granules and asphalt binder [27]. In the present work, granulated non-tire automotive rubber crumbs had a higher density (1.16) than that of PPO (0.85) and base asphalt (1.03). The density of the binder R (with 16% rubber alone) was 1.09, whereas the density of the rubberized binder RP6p with pre-swelled rubber (16% rubber and 6% PPO) was 1.04. A reduction in density implies that pre-swelled rubber granules with PPO are able to adjust the dynamics of the rubberized asphalt matrix and reduce the density differential between the rubber modifier and the base asphalt, therefore improving the compatibility of rubberized asphalts during pro-longed thermal storage.

Moreover, the extent of rubber swelling affects its degradation and consequently its solubility in the asphalt matrix, which impacts its storage stability. A typical rubberized asphalt usually reaches its swelling equilibrium after a prolonged period (typically more than 5 hours) of conditioning and agitation as the typical process of rubber swelling in binder is one of selective absorption mechanism constrained by the high-molecular-weight components in the base asphalt [55]. The PPO with higher amounts of low-molecular-weight components propagates into the internal structures of rubber during pre-mixing and thermal conditioning and expedites the swelling of rubber granules in the asphalt matrix. As a result, the integration of PPO into rubber particles develops a new paradigm for the subsequent disintegration of rubber granules in the asphalt phase along with an appreciable rubber swelling, and thus improves the dispersion of rubber particles in the asphalt matrix, and increases the storage stability.

Further, the improved storage stability of non-tire automotive rubber modified binders following the inclusion of sulfur in both sequential and pre-swelling methods could be ascribed to its crosslinking capabilities. Sulfur retards phase separation tendency and enhances thermal storage stability by crosslinking unsaturated bonds in polymers and asphalt hydrocarbons by creating covalent bonds through sulfide and/or polysulfide bonds [56]. EPDM rubber derived from non-tire automotive parts possesses a side-chain pendant with unsaturated double bonds, allowing the sulfur to perform as an efficient crosslinking additive to bond rubber and asphalt phases [57]. This leads to the improved storage stability of sulfur-containing binders at each PPO dosage. Sulfur as a crosslinking additive, on the other hand, had a reduced influence on storage stability performance after prolonged storage (96 hours) at high temperatures. This can be explained in terms of the breakdown of crosslinking networks as a result of the detrimental effect of aging caused by long-term storage at high temperatures [58]. However, the effectiveness of PPO and the pre-swelling approach in the substantial increment in the compatibility of non-tire automotive rubberized asphalt was still prevalent after the prolonged thermal storage of 96 hours, as demonstrated by the analysis of storage stability performance using conventional, chemical, microstructural, and rheological evaluations.

## Conclusions

The study aimed to assess the storage stability of composite asphalt binders using non-tire automobile rubber (EPDM rubber) and PPO, as well as the addition of a crosslinking agent (sulfur). Two different approaches were used to prepare composite rubberized binders: (1) sequential incorporation of PPO and granulated rubber crumbs to the base asphalt binder and (2) inclusion of rubber granules pre-swelled with PPO at 90°C to neat asphalt. Based on the binder formulation methods and the addition of sulfur, four different categories of binders were prepared, namely sequential (SA), sequential with sulfur (SA-S), pre-swelling (PA), and pre-swelling with sulfur (PA-S). The binders were subjected to thermal storage for 48 and 96 hours. In total, 34 rubberized binders were evaluated for storage stability based on various separation indices (SIs). Based on the observations from the experimental approach employed in this study, the following conclusions can be derived:

- Various separation indices revealed that incorporation of PPO for composite modification of rubberized asphalt binder improves the storage stability. Both sequential and pre-swelling approaches exhibited an improvement in SIs with an increment in PPO contents, however, pre-swelling significantly outperformed the sequential method. When compared to the observations on binders subjected to 48 hours storage, the storage stability performance for all asphalts decreased after 96 hours of thermal storage.
- The application of sulfur enhanced the SIs of composite rubberized asphalts at each PPO dosage and both binder preparation approaches (sequential and pre-swelling) when subjected to 48 hours of thermal storage; however, prolonged thermal storage for 96 hours at high temperature had no apparent effect on storage stability performance owing to the breakdown of developed crosslinking networks as a result of the detrimental effect of aging.
- FTIR spectroscopy revealed that composite rubberized binders produced using the pre-swelling method had better spectral proximity than those prepared with the sequential approach. Furthermore, the *SI*_*FTIR*_ for the pre-swelling approach was lower than the sequential approach based on the absorbance differential corresponding to the distinctive peak of EPDM, and the *SI*_*FTIR*_ was further lowered with the addition of sulfur.
- The higher dissimilarities between AFM micrographs for the two sub-sections of the storage tube of the control rubberized asphalt with non-tire automotive EPDM rubber alone, extracted after thermal storage, can be attributed to the greater quantum of granulated rubber crumbs present in the bottom sub-section. The topographic images for asphalt from the two sub-sections showed substantial resemblance with smaller catana domains for the composite rubberized binder prepared with the pre-swelling approach, indicating the improved stability of the composite rubberized binder during thermal storage.
- The *SI*_*RET*_, defined as the mass difference of extracted undissolved rubber particles from the top and bottom sections, decreased with increasing PPO dosages for both sequential and pre-swelling approaches after 48 and 96 hours of thermal storage. This indicates that increase in the PPO dosage improved the compatibility of composite rubberized asphalts. The PA-S and PA approaches showed lower *SI*_*RET*_ results than sequential approach indicating that the advantages of pre-swelling the rubber with PPO remained even after 96 hours of extended thermal storage.
- The best storage stability performance in both sequential and pre-swelling methods with and without sulfur was achieved for an optimal PPO dosage of 6% with a non-tire automotive rubber (EPDM rubber) dosage of 16% in asphalt binder composite modification. Furthermore, the separation indices based on chemical analysis *(SI*_*FTIR*_*)* and rubber extraction test *(SI*_*RET*_) showed a better correlation with rheology-based separation index (*SI*_G*/sin δ_) than the conventional storage stability indicator (SPD).

## Future perspective

The outcomes of the study suggest that composite modifications of base asphalt with PPO and non-tire automotive EPDM rubber can be achieved with the desired storage stability. Pre-swelling of rubber with PPO via thermal conditioning prior to addition with a base asphalt improves its performance significantly in terms of storage stability. Blending oil obtained through pyrolysis of waste plastic with waste EPDM rubber from non-tire automotive components is a critical approach for achieving sustainability in the synthesis of rubberized asphalts. Studies should be also conducted on the characteristic evaluation of composite rubberized asphalts against thermal and ultraviolet aging. Further studies involving characterization of asphalt mixes prepared with composite rubberized binder having EPDM rubber and PPO will be quite important. A systematic environmental and economic analysis of the formulation of the composite rubberized asphalt using waste automotive derived EPDM rubber and plastic pyrolytic oil should also be performed.

## Supporting information

S1 File (ZIP)
